# Utility of Immunophenotypic Measurable Residual Disease in Adult Acute Myeloid Leukemia—Real-World Context

**DOI:** 10.3389/fonc.2019.00450

**Published:** 2019-06-13

**Authors:** Nikhil Patkar, Chinmayee Kakirde, Prasanna Bhanshe, Swapnali Joshi, Shruti Chaudhary, Yajamanam Badrinath, Sitaram Ghoghale, Nilesh Deshpande, Shraddha Kadechkar, Gaurav Chatterjee, Sadhana Kannan, Dhanalaxmi Shetty, Anant Gokarn, Sachin Punatkar, Avinash Bonda, Lingaraj Nayak, Hasmukh Jain, Bhausaheb Bagal, Hari Menon, Manju Sengar, Syed Hasan Khizer, Navin Khattry, Prashant Tembhare, Sumeet Gujral, Papagudi Subramanian

**Affiliations:** ^1^Haematopathology Laboratory, ACTREC, Tata Memorial Centre, Mumbai, India; ^2^Biostatistics, ACTREC, Tata Memorial Centre, Mumbai, India; ^3^Department of Cytogenetics, ACTREC, Tata Memorial Centre, Mumbai, India; ^4^Adult Haematolymphoid Disease Management Group, Tata Memorial Centre, Mumbai, India; ^5^Haemato-Oncology, CyteCare Cancer Hospital, Bangalore, India

**Keywords:** measurable residual disease, acute myeloid leukemia, FCM MRD, real-world data AML, AML MRD

## Abstract

**Introduction:** One of the mainstays of chemotherapy in acute myeloid leukemia (AML) is induction with a goal to achieve morphological complete remission (CR). However, not all patients by this remission criterion achieve long-term remission and a subset relapse. This relapse is explained by the presence of measurable residual disease (MRD).

**Methods:** We accrued 451 consecutive patients of adult AML (from March 2012 to December 2017) after informed consent. All patients received standard chemotherapy. MRD testing was done at post-induction and, if feasible, post-consolidation using 8- and later 10-color FCM. Analysis of MRD was done using a combination of difference from normal and leukemia-associated immunophenotype approaches. Conventional karyotyping and FISH were done as per standard recommendations, and patients were classified into favorable, intermediate, and poor cytogenetic risk groups. The presence of *FLT3*-ITD, *NPM1*, and *CEBPA* mutations was detected by a fragment length analysis-based assay.

**Results:** As compared to Western data, our cohort of patients was younger with a median age of 35 years. There were 62 induction deaths in this cohort (13.7%), and 77 patients (17.1%) were not in morphological remission. The median follow-up was 26.0 months. Poor-risk cytogenetics and the presence of *FLT3*-ITD were significantly associated with inferior outcome. The presence of post-induction MRD assessment was significantly associated with adverse outcome with respect to OS (*p* = 0.01) as well as RFS (*p* = 0.004). Among established genetic subgroups, detection of MRD in intermediate cytogenetic and *NPM1* mutated groups was also highly predictive of inferior outcome. On multivariate analysis, immunophenotypic MRD at the end of induction and *FLT3*-ITD emerged as independent prognostic factors predictive for outcome.

**Conclusion:** This is the first data from a resource-constrained real-world setting demonstrating the utility of AML MRD as well as long-term outcome of AML. Our data is in agreement with other studies that determination of MRD is extremely important in predicting outcome. AML MRD is a very useful guide for guiding post-remission strategies in AML and should be incorporated into routine treatment algorithms.

## Introduction

Acute myeloid leukemia (AML) is a biologically heterogeneous disease in which the karyotype is highly predictive of outcome (1). Chromosomal translocations resulting in chimeric gene fusions such as *RUNX1-RUNX1T1, CBFB-MYH11, KMT2A, BCR-ABL1, DEK-NUP214, NUP98-NSD1, GATA2*, and *MECOM* are the important determinants of outcome in AML (2). The last few years have seen an explosion of information in terms of newer gene mutations in AML that affect diverse biological pathways. Some of these genes are of prognostic relevance and may offer newer avenues for risk stratification of AML (3–10). The presence of mutations in genes encoding for activated signaling pathways (e.g., *KIT* and *FLT3*), or *NPM1*, chromatin–spliceosome complex, tumor suppressor genes (such as *TP53* and *WT1*) and transcription factors (such as *RUNX1* and *CEBPA*), and cohesion complex are increasingly recognized as important genetic alterations in AML (2). Patients who bear favorable risk mutations (*NPM1* and *CEBPA*) do not benefit from intensive therapeutic regimens such as allogeneic bone marrow transplantation (aBMT), as this has a transplant-related mortality risk of 10–20% (11). Patients who harbor unfavorable mutations such as *FLT3*-internal tandem duplications (*FLT3*-ITD) are less than likely to attain long-term disease-free survival unless treated with intense regimens (11).

In addition to cytogenetics and molecular risk stratification, early response to chemotherapy is an important variable. Traditionally, evaluation of this remission has been done by light microscopy, where morphological remission is defined by <5% blasts in the post-treatment bone marrow (12). The detection of residual disease by techniques more sensitive than light microscopy is called measurable residual disease (MRD) and is an important prognostic marker, which dictates outcome of disease. Importantly, detectable MRD is a measure of impending relapse and offers a therapeutic window to modify treatment to prevent overt relapse or de-intensify treatment. For AML with recurrent cytogenetic abnormalities as well as *NPM1* gene mutations, real-time PCR is the most efficient method of detecting the tumor burden. For patients who do not harbor these abnormalities, immunophenotyping is the most accepted and established technique to detect MRD (13, 14). Immunophenotyping-based AML MRD (FCM-MRD) is conceptually different from MRD detection in B or T-ALL. This is because of a lack of common cell surface antigens or a uniform definition for abnormal myeloid blasts. Even so, with knowledge of normal regenerative hematopoiesis and use of carefully selected antibody combinations that characterize myeloid maturation from normal progenitors as well as detect leukemia-associated immunophenotype (LAIP), FCM-MRD can be reliably performed (15). The clinical significance of MRD in AML has been proved in numerous studies. Earlier studies by San Miguel et al. demonstrated the utility of post-induction MRD in risk stratification of AML (16, 17). Following this, a number of studies have been published across Europe and North America that have established the role of MRD in AML as a prognostic and predictive marker for children as well as adults (18–26). Based on these data, it seems that morphologic estimation of blasts to evaluate response to chemotherapy is a thing of the past and MRD-based risk assessment is ready for primetime in AML (27). There have been very few studies on MRD in acute lymphoblastic leukemia from India (28) and none on AML. Furthermore, very few studies have been published from developing countries on FCM-MRD in AML. From a resource-constrained setting such as India, conventional treatment strategies of AML therapy, which include bone marrow transplant, are beyond the reach of majority of patients due to socioeconomic considerations. So, there is a clinical need to identify patients with good prognosis to target scarce available resources effectively. With that goal in mind, this lacuna in literature needs to be addressed.

In this manuscript, which is a first from India, we describe a single center experience on FCM-MRD for AML. We also describe the follow-up details of these patients and describe how MRD contributes to early determination of relapse.

## Materials and Methods

### Patient Accrual and Initial Work Up

The protocol was approved by the institutional ethics committee. We accrued all consecutive adult (>18 years) patients of *de novo* AML who consented to being a part of the study and who received standard chemotherapy from March 2012 to December 2017. In all patients, the diagnosis of AML was made as per WHO 2008 recommendations. Conventional karyotyping and FISH were performed as per standard recommendations (29).

### Treatment Protocols and Sampling for MRD

All patients received “3+7” induction therapy with daunorubicin (60 mg/m^2^ D1–D3) and cytarabine (100 mg/m^2^/day continuous infusion D1–D7). We initially defined complete remission (CR) and CR with incomplete hematological recovery (CRi) as per European LeukemiaNet (ELN) recommendations. However, as we could not detect any clinical relevance of this distinction in our cohort (OS (*p* = 0.4) and RFS (*p* = 0.8), we then defined CR as a morphologic leukemia-free state. Thus, CR was defined as per morphology (<5% blasts in bone marrow/absence of circulating blasts and blasts with Auer rods) at the end of induction. If they achieved CR, they received three courses of 12–18 g/m^2^ high-dose cytarabine (HiDAC) or underwent allogeneic transplant if feasible. BM aspiration was performed 21–28 days after the start of induction chemotherapy for MRD and morphology. If BM showed morphological remission (i.e., normocellular marrow with <5% blasts and normal peripheral blood counts), then patients received first post-remission consolidation therapy with 12–18 g/m^2^ of cytosine arabinoside (HiDAC) over a 5-day period. BM aspiration was repeated 21–28 days after first consolidation chemotherapy for MRD and morphology.

### Detection of Gene Mutations

Genomic DNA was extracted from bone marrow or peripheral blood using commercial silica membrane-based columns (QIAamp DNA Blood Mini Kit, Qiagen, Germany) and was used for downstream applications.

- *FLT3-ITD and NPM1 mutation detection*: A multiplex PCR procedure was used, with fluorescently labeled (FAM, NED) primer combinations that detected the FLT3-ITD and *NPM1* insertion mutations. Amplicons generated by *FLT3* and *NPM1* primers spanned the commonly occurring ITD regions and the common type A–F mutations (30).
- *CEBPA*: Mutation analysis for this gene was approached by fragment length analysis as well (31). Fluorescently labeled primers flanking *TAD1* and *ZIP* domains were multiplexed in a single PCR reaction, whereas a separate reaction amplified the *TAD2* domain. PCR was followed by capillary electrophoresis on an ABI3500 genetic analyzer.

### Immunophenotypic Assessment of MRD

Our approach to detection of immunophenotypic MRD detection has been published previously (32). The analysis was performed as follows: Familiarity with normal myeloid maturation and standardization of templates were achieved on stressed regenerative bone marrows (e.g., ALL post-induction that were MRD negative) using the antibody panels described previously (32). Patients accrued from July 2012 to February 2015 (138 patients) were processed using a three-tube, eight-color MRD assay. Subsequently, patient samples of 225 patients were processed using a two-tube, 10-color MRD assay. Identical panel was used for diagnostic sample, post-induction, and post-consolidation. A total of 500,000 events were acquired per tube with the three-tube assay, and 1.6 million events per tube were obtained per tube with the two-tube, 10-color assay. Analysis of MRD was done using Kaluza 1.3 by a combination of difference from normal approach that focused on the development of myeloid progenitors to mature cells and LAIP approaches. Any detectable MRD was called as MRD positive, and MRD was calculated as a percentage of abnormal leukemic cells per total nucleated cells. To demonstrate sensitivity of the MRD assay, we performed a linearity experiment where we serially diluted an OCIAML3 cell line in a normal bone marrow. The results for the 10-color assay have been published recently (32). To demonstrate that the eight-color assay has a comparable sensitivity, we performed a similar experiment where the OCIAML3 cell line was diluted in normal bone marrow. We could demonstrate that the eight-color assay has a similar sensitivity of 0.01% (see Supplementary Data Sheet).

### Evaluation of Treatment Outcome

**Figure 1:**
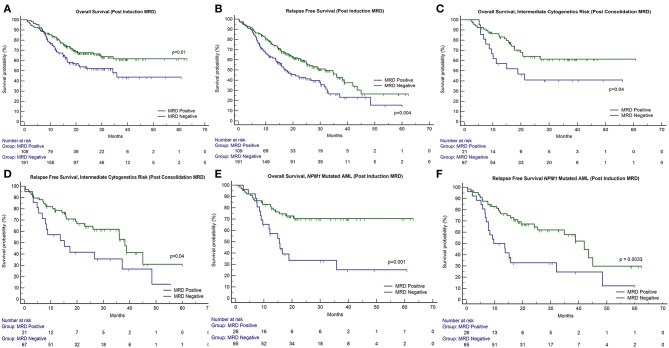
Impact of response after induction and consolidation phases of chemotherapy on outcome in patients of acute myeloid leukemia. OS, overall survival; RFS, relapse-free survival. **(A,B)** The presence of post-induction MRD assessment was significantly associated with adverse outcome with respect to OS (*p* = 0.01) as well as RFS (*p* = 0.004). **(C,D)** The presence of post-induction MRD was predictive of an inferior OS in patients who were in intermediate cytogenetic risk-group as well as RFS. **(E,F)** Similarly, patients with NPM1 mutations harboring MRD at the end of induction (*n* = 26 out of 91 cases, 28.6%) were associated with inferior OS (*p* = 0.001) as well as RFS (*p* = 0.003).

Overall survival (OS) was calculated from date of diagnosis to time of last follow-up or death. Relapse-free survival (RFS) was calculated from date of CR until time to relapse or death or last follow-up if in CR. Results of the MRD assays, cytogenetic and molecular risk groups were analyzed for their impact on OS and RFS using log-rank test (33). Statistical analysis was done using MedCalc 14.8. Univariate analysis of the MRD assays and cytogenetic and molecular risk groups was analyzed for their impact on OS and RFS using the Kaplan–Meier technique and compared using log-rank test (Figure 1F). All variables found significant in the univariate analysis were considered for multivariate analysis. A Cox proportional hazards regression model was used for multivariate analysis to assess the effect of risk factors on the OS and RFS. Separate Cox regression models were reported for post-induction and consolidation sampling of MRD results.

### *NPM1* NGS MRD

Our strategy for *NPM1* MRD using ultradeep sequencing has been described recently (32). Briefly, Illumina adapter-linked locus-specific primers were incorporated in a one-step PCR reaction along with sample-specific dual indices using 600 ng of DNA as template. Data were analyzed using PANDAseq, bwa v0.7.12, Samtools v0.1.19, and VarScan v.2.3.7. The assay was validated to have a sensitivity of 0.001%. *NPM1* NGS MRD was measured as a 1-log reduction between PI and PC time points. Here, patients with <1-log reduction were classified as *NPM1* NGS MRD positive and those with >1-log reduction were classified as *NPM1* NGS MRD negative. In the present manuscript, we specifically combined the two modalities of MRD detection (NGS and FCM), where patients classified as dual MRD negative were compared to the rest.

### Differences in Relapse Characteristics of Patients Where Post-induction MRD Was Assessed

To determine any differences in PI MRD-positive patients who relapsed and those who did not relapse, we compared the baseline characteristics of these groups for *FLT3*-ITD, *NPM1, CEBPA*, and cytogenetic risk using chi-squared test. A similar analysis was done to ascertain differences in PI MRD-negative patients.

## Results

A summary of clinical and laboratory parameters can be appreciated in an overview in Table 1.

**Table 1:** Demographic and clinical characteristics of patients.

**Parameter**	**Observation (%)**
**Demographics**	
Age	Range: 18–63 years; median: 35 years
Sex	Male:female: 1.6:1
**Clinical characteristics**	
Total number of patients accrued	451
Induction deaths	62
Refractory cases	77
**Remission characteristics**	
Complete remission (CR)	58
CR with incomplete hematologic recovery (CRi)	254
**Laboratory characteristics (*****n*** **=** **312)**	
**Cytogenetics**	
1. Favorable risk cytogenetics	110 (35.3%)
2. Intermediate-risk cytogenetics^*^	164 (52.6%)
3. Poor-risk cytogenetics	38 (12.2%)
**Molecular testing**	
1. *FLT3*-internal tandem duplication	68 positive out of 311 tested (21.9%)
2. *NPM1* mutation	92 positive out of 311 tested (29.9%)
3. *CEBPA* mutation	24 positive out of 293 tested (8.2%)
**FCM—measurable residual disease**	
1. Post-induction MRD measurements	300
a. MRD positive	109 positive out of 300 (36.3%)
b. MRD negative	191 negative (63.7%)
2. Post-consolidation MRD measurements	188
a. MRD positive	48 positive out of 188 (25.5%)
b. MRD negative	140 negative (74.5%)
3. Interaction between MRD measurements	
a. Paired (PI and PC) MRD measurements	183 (58.7%) out of 312
b. MRD positive at all measurements (PI and PC)	41 (22.4%)
c. PI MRD positive; negative subsequently	40 (21.9%)
d. PC MRD positive; negative at PI time point	4 (2.2%)
e. Negative at all time points	98 (53.6%)

* *Includes patients that could not be classified as either favorable or poor risk due to metaphase failures*.

### Patient Accrual and Morphological Assessment of Response to Chemotherapy

A total of 451 patients were accrued in the study over a period spanning 6 years. In contrast to Western studies, most of the patients in our cohort were young with a median age of 35 years (age ranging from 18 to 63 years), with a slight male predominance (M/F = 1.6:1). There were 62 induction deaths in our patient cohort, and additionally, 77 patients were not in morphological remission. After exclusion of these patients, a total of 312 patients remained. The subsequent analysis refers to these 312 patients.

### Clinical Outcome

The median follow-up was 26.0 months. The survival analysis indicated that the mean OS was 41.4 months (median not reached; 95% CI: 38.2–44.6) and median RFS was 26 months (95% CI: 20.3–32.1). Only 20 patients (6.4%) out of the 312 analyzed here received allogeneic bone marrow transplantation. There was no difference in outcome for OS (*p* = 0.2) and RFS (*p* = 0.3) between patients who underwent transplantation in comparison to patients who did not undergo it. As compared to Western data, this lack of difference in survival is presumably due to the very small numbers of patients in the transplant group. This is typical of a resource-constrained setting.

### Cytogenetics and Gene Mutations

**Figure 2:**
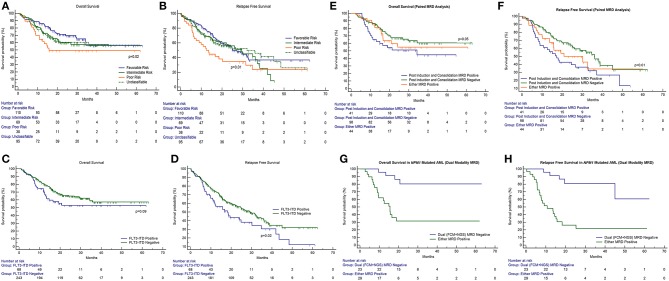
Impact of cytogenetics, *FLT3*-internal tandem duplication, paired time point (PI and PC) FCM MRD analysis, and combined modality (FCM + NGS) MRD in *NPM1* mutated AML on outcome in patients of acute myeloid leukemia. OS, overall survival; RFS, relapse-free survival. **(A,B)** The presence of poor-risk cytogenetics predicted for an inferior OS (*p* = 0.02) as well as RFS (*p* = 0.01) as compared to favorable risk. **(C,D)** The presence of FLT3-ITD predicted for an inferior OS (*p* = 0.09) and RFS (*p* = 0.02). **(E,F)** Patients with detectable MRD at both time points had an inferior OS [median OS: 32.3 months; 95% CI (13.1–34.3 months); *p* = 0.05] and RFS [median RFS: 17.3 months; 95% CI (8.9–32.3 months); *p* = 0.01] as compared to patients who were MRD negative at both time points [median not reached] and RFS [median RFS: 36.0 months; 95% CI (26.0–45.0 months)]. **(G,H)** For NPM1 mutated patients, who were MRD negative by both FCM and NGS had a far superior OS (*p* = 0.002) and RFS (*p* < 0.001) as compared to other patients.

Patients could be classified as favorable cytogenetic risk (*n* = 110, 35.3%), intermediate risk (*n* = 69, 22.1%), and poor risk (*n* = 38, 12.2%). We were unable to classify 95 patients because of metaphase failures (30.4%). The presence of poor-risk cytogenetics predicted for an inferior OS (*p* = 0.02) as well as RFS (*p* = 0.01) as compared to favorable risk (Figures 2A,B). *FLT3*-ITD was harbored by 21.9% of 311 patients tested (*n* = 68). The presence of *FLT3*-ITD predicted for an inferior OS (*p* = 0.09) and RFS (*p* = 0.02) as can be seen in Figures 2C,D. Among patients who were neither favorable nor high-risk cytogenetics, the presence of *FLT3*-ITD showed a tendency to predict for inferior OS (*p* = 0.09) and RFS (*p* = 0.08). *NPM1* (*n* = 92 positive cases) and *CEBPA* (*n* = 24 positive cases) mutations were detected in 29.9% and 8.2% of patients, respectively, in various combinations. Neither *NPM1* nor *CEBPA* gene mutations had any bearing on outcome, with respect to RFS or OS. This was the case even when the intermediate cytogenetic risk group was separately analyzed for the prognostic impact of gene mutations.

### Prevalence of MRD

Post-induction MRD sampling was done in 300 patients, out of which MRD could be detected in 109 patients (36.3%) at a range from 0.02% to 17.3% (median = 0.9%). Post-consolidation MRD results were available in 188 samples, out of which MRD was detected in 48 (25.5%) ranging from 0.002 to 7.7% (median = 0.3%). The presence of post-induction MRD assessment was significantly associated with adverse outcome with respect to OS (*p* = 0.01) as well as RFS (*p* = 0.004) as seen in Figures 1A,B. However, the presence of MRD at the end of consolidation was not associated with an adverse outcome with respect to OS (*p* = 0.16) and RFS (*p* = 0.09). These details can be appreciated in Tables 1–3.

**Table 2:** Prognostic significance of MRD, gene mutations, and cytogenetics in AML by univariate Cox analysis.

**Univariate Cox analysis**				
	**Overall survival (OS)**		**Relapse-free survival (RFS)**	
	**HR (95% CI)**	***P***	**HR (95% CI)**	***P***
**CYTOGENETICS**				
Favorable risk	1	0.024	1	0.05
Poor risk	1.99 (1.02–3.72)		1.73 (1.01–2.96)	
***FLT3*****-INTERNAL TANDEM DUPLICATION (*****n*** **=** **311)**				
Wild type	1	0.09		0.02
Mutated	1.44 (0.9–2.3)		1.48 (1.0–2.18)	
***NPM1*** **(*****n*** **=** **311)**				
Wild type	1		1	0.33
Mutated	1.06 (0.7–1.6)		0.85 (0.6–1.2)	
***CEBPA*** **(*****n*** **=** **293)**				
Wild type	1	0.9		0.8
Mutated	1.1 (0.5–2.1)		1.1 (0.6–1.9)	
**POST-INDUCTION MRD (*****n*** **=** **300)**				
MRD negative	1	0.02		0.004
MRD positive	1.6 (1.1–2.3)		1.6 (1.1–2.2)	
**POST-CONSOLIDATION MRD (*****n*** **=** **188)**				
MRD negative	1	0.16		0.09
MRD positive	1.4 (0.8–2.5)		1.4 (0.9–2.3)	
POST-INDUCTION mrd INTERMEDIATE CYTOGENETIC RISK^*^ **(*****n*** **=** **161)**				
MRD negative	1	0.02	1	0.04
MRD positive	1.8 (1.1–3.2)		1.6 (1.0–2.5)	
POST-CONSOLIDATION mrd INTERMEDIATE CYTOGENETIC RISK^*^				
**(*****n*** **=** **88)**				
MRD negative	1	0.04		0.04
MRD positive	2.0 (0.9–4.6)		1.9 (0.9–3.8)	
**POST-INDUCTION mrd npm1 MUTATED AML (*****n*** **=** **91)**				
MRD negative	1	0.001	1	0.0033
MRD positive	2.9 (1.3–6.2)		2.3 (1.2–4.6)	
**POST-CONSOLIDATION mrd npm1 MUTATED AML (*****n*** **=** **49)**				
MRD negative	1	0.06	1	0.06
MRD positive	2.5 (0.6–9.9)		2.3 (0.7–7.6)	
**POST-INDUCTION MRD NPM1+FLT3-ITD MUTATED AML (*****n*** **=** **38)**				
MRD negative	1	0.02	1	0.14
MRD positive	3.2 (0.9–10.9)		1.9 (0.7–5.1)	
**EITHER (FCM or NGS) MRD: NPM1+** **MUTATED AML (*****n*** **=** **54)**				
MRD negative	1	0.0002	1	<0.0001
MRD positive	6.1 (2.7–13.8)		6.0 (2.8–12.9)	

*ITD, internal tandem duplication; MRD, measurable residual disease*.

* *Cases that were neither favorable nor poor-risk cytogenetics. OS, overall survival; RFS, relapse-free survival; HR, hazards ratio; CI, confidence interval*.

**Table 3:** Difference in overall survival and relapse-free survival between MRD-positive and MRD-negative groups.

	**Overall survival (OS)**		**Relapse-free survival (RFS)**	
**Post-induction MRD**				
MRD negative	Mean OS: 42.8 months; 95% CI (39.0–46.5 months)	***p=0.02***	Mean RFS: 32.1 months; 95% CI (28.3–35.9 months)	***p=0.004***
	Median OS: not reached		Median RFS: 31.8 months; 95% CI (24.8–38.8 months)	
MRD positive	Mean OS: 35.5 months; 95% CI (30.0–41.1 months)		Mean RFS: 25.0 months; 95% CI (20.4–29.7 months)	
	Median OS: 34.3 months; 95% CI (16.5–35.8 months)		Median RFS: 17.3 months; 95% CI (13.7–26.6 months)	
**Post-consolidation MRD**				
MRD negative	Mean OS: 41.2 months; 95% CI (37.1–45.2 months)	*p = 0.16*	Mean RFS: 33.3 months; 95% CI (29.2–37.5 months)	*p = 0.09*
	Median OS: not reached		Median RFS: 34.7 months; 95% CI (25.3–45.0 months)	
MRD positive	Mean OS: 36.3 months; 95% CI (29.1–43.5 months)		Mean RFS: 28.1 months; 95% CI (21.4–34.7 months)	
	Median OS: 12.1 months; 95% CI (8.8–20.4 months)		Median RFS: 18 months; 95% CI (10.7–37.1 months)	
**Post-induction MRD intermediate cytogenetic risk**				
MRD negative	Mean OS: 33.5 months; 95% CI (30.0–37.0 months)	***p=0.02***	Mean RFS: 28.4 months; 95% CI (25.0–31.7 months)	***p=0.04***
	Median OS: not reached		Median RFS: 36.0 months; 95% CI (24.8–41.9 months)	
MRD positive	Mean OS: 32.4 months; 95% CI (25.3–39.6 months)		Mean RFS: 24.0 months; 95% CI (17.9–30.2 months)	
	Median OS: 18.9 months; 95% CI (12.9–35.8 months)		Median RFS: 15.1 months; 95% CI (9.2–29.9 months)	
**Post-consolidation MRD intermediate cytogenetic risk**				
MRD negative	Mean OS: 42.5 months; 95% CI (36.4–48.6 months)	***p=0.04***	Mean RFS: 35.3 months; 95% CI (28.8–41.7 months)	***p=0.04***
	Median OS: not reached		Median RFS: 38.2 months; 95% CI (26.0–45.0 months)	
MRD positive	Mean OS: 29.3 months; 95% CI (19.4–32.2 months)		Mean RFS: 23.5 months; 95% CI (14.7–32.3 months)	
	Median OS: 18.9 months; 95% CI (9.6–21.3 months)		Median RFS: 15.1 months; 95% CI (7.9–37.1 months)	
**Post-induction MRD NPM1 mutated AML**				
MRD negative	Mean OS: 46.7 months; 95% CI (39.7–51.6 months)	***p=0.001***	Mean RFS: 35.7 months; 95% CI (29.5–41.9 months)	***p=0.003***
	Median OS: not reached		Median RFS: 41.9 months; 95% CI (25.3–45.0 months)	
MRD positive	Mean OS: 25.9 months; 95% CI (16.5–35.4 months)		Mean RFS: 21.7 months; 95% CI (13.1–30.3 months)	
	Median OS: 15.5 months; 95% CI (9.6–35.8 months)		Median RFS: 9.7 months; 95% CI (7.7–32.1 months)	

*OS, overall survival; RFS, relapse-free survival; CI, confidence interval. The parameters with statistically significant P-values are highlighted in bold*.

### Correlation of MRD With Specific Risk Groups

We then examined whether the assessment of MRD in individual cytogenetic and molecular risk groups was predictive of outcome. The presence of post-induction MRD was predictive of an inferior OS in patients who were neither favorable nor poor-risk cytogenetic risk (*p* = 0.01) as well as RFS (*p* = 0.04). Similarly, the presence of MRD at the end of consolidation was associated with inferior OS as well as RFS (*p* = 0.04 each) as seen in Figures 1C,D. MRD at either time points was not predictive of outcome in favorable or poor-risk cytogenetic AML. We then assessed the clinical relevance of MRD in *NPM1* mutated AML. We found a strong correlation of MRD and outcome in this subgroup (Table 3). Patients with *NPM1* mutations harboring MRD at the end of induction (*n* = 26 out of 91 cases, 28.6%) were associated with inferior OS (*p* = 0.001) as well as RFS (*p* = 0.003) as seen in Figures 1E,F. A similar trend was seen at the end of consolidation for OS and RFS (*n* = 7 out of 49 cases, 8.2%, *p* = 0.06). We further assessed the relevance of MRD at the end of induction in *NPM1* and *FLT3*-ITD mutated AML (*n* = 38). Here, the presence of MRD (*n* = 10, 26.3%) was significantly predictive of an inferior outcome for OS (*p* = 0.02) but not RFS (*p* = 0.14). Multivariate models included cytogenetics, FLT3-ITD, and MRD. Separate models were considered for PI and PC MRD. On multivariate analysis, both immunophenotypic MRD (end of induction) and *FLT3*-ITD emerged as independent prognostic factors for RFS (*p* = 0.003) as seen in Table 4. For OS, only the presence of end induction MRD mattered in the multivariate model (Table 4).

**Table 4:** Prognostic significance of MRD, gene mutations, and cytogenetics in AML by multivariate Cox analysis.

**MRD time point**	**Factors**	**HR**	**95% CI**	***p*-value**
**MULTIVARIATE COX ANALYSIS: OVERALL SURVIVAL**				
Post-induction MRD	Cytogenetic risk—Intermediate risk	1.43	0.91–2.25	0.12
	Cytogenetic risk—Poor risk	1.74	0.93–3.24	0.08
	**Post-induction MRD positive**	**1.62**	**1.1–2.37**	**0.015**
	FLT3-internal tandem duplication positive	1.4	0.9–2.2	0.15
Post-consolidation MRD	Cytogenetic risk—Intermediate risk	1.55	0.89–2.7	0.12
	Cytogenetic risk—Poor risk	2.1	1.0–4.4	0.06
	Post-consolidation MRD positive	1.41	0.84–2.4	0.19
	FLT3-internal tandem duplication positive	1.31	0.7–2.55	0.4
**MULTIVARIATE COX ANALYSIS: RELAPSE-FREE SURVIVAL**				
Post-induction MRD	Cytogenetic risk—Intermediate risk	1.14	0.79–1.64	0.5
	Cytogenetic risk—Poor risk	1.41	0.84–2.4	0.2
	**Post-induction MRD positive**	**1.61**	**1.17**–**2.22**	**0.003**
	**FLT3-internal tandem duplication positive**	**1.51**	**1.04–2.2**	**0.03**
Post-consolidation MRD	Cytogenetic risk—Intermediate risk	1.11	0.70–1.75	0.7
	Cytogenetic risk—Poor risk	1.52	0.80–2.89	0.2
	Post-consolidation MRD positive	1.4	0.90–2.17	0.14
	FLT3-internal tandem duplication positive	1.42	0.80–2.51	0.14

*ITD, internal tandem duplication; MRD, measurable residual disease; OS, overall survival; RFS, relapse-free survival; HR, hazards ratio; CI, confidence interval. The parameters with statistically significant P-values are highlighted in bold*.

### Interaction Between MRD Time Points

A total of 183 paired MRD samples were analyzed, where FCM-MRD assessment was done at both PI and PC time points. These results can be appreciated in Table 1 and Figures 2E,F. Patients with detectable MRD at both time points had an inferior OS [mean OS: 33.9 months; 95% CI (26.1–41.6 months), median OS: 32.3 months; 95% CI (13.1–34.3 months); *p* = 0.05] and RFS [mean RFS: 24.6 months; 95% CI (18.0–31.2 months), median RFS: 17.3 months; 95% CI (8.9–32.3 months); *p* = 0.01] as compared to patients who were MRD negative at both time points [mean OS: 39.3 months; 95% CI (35.0–43.6 months), median not reached] and RFS [mean RFS: 33.1 months; 95% CI (28.8–37.3 months), median RFS: 36.0 months; 95% CI (26.0–45.0 months)].

### NPM1 NGS MRD

Poor correlation of values was found when single time point *NPM1* NGS MRD measurements (for example, *NPM1* NGS MRD measured at PI time point) were compared to FCM MRD (32). We then assessed *NPM1* NGS MRD in 54 cases (where paired samples were available and MRD was defined by log reduction values) in combination with FCM-MRD. Here, we determined that patients who were dual MRD negative had a far superior OS (*p* = 0.002) and RFS (*p* < 0.001) as compared to other patients [for dual MRD-negative group; mean OS: 53.8 months; 95% CI (45.6–62.0 months), median not reached; mean RFS: 49.8 months; 95% CI (40.3–59.3 months), median not reached; for either MRD-positive group; mean OS: 26.9 months; 95% CI (17.7–36.0 months), median OS: 15.5 months; 95% CI (9.4–18.9 months); mean RFS: 20.9 months; 95% CI (7.0–15.7 months)]. These results are seen in Table 2 and Figures 2G,H.

### Differences in Relapse Characteristics of Patients Where Post-induction MRD Was Assessed

No differences were found in characteristics of patients who were MRD positive and relapsed/did not relapse. Similarly, there were no differences in patients who were MRD negative. These results can be seen in Supplementary Data Sheet.

## Discussion

There have been a number of recently published papers that have specifically addressed issues pertaining to the treatment of AML in a resource-constrained setting (34–42). What is common to these studies is a younger patient cohort, higher percentage of deaths during induction, and survival metrics that are inferior when compared to western data. Higher mortality during induction in these cohorts has been attributed to suboptimal infrastructure in hospitals resulting in fungal and bacterial infections, which are often multi-drug resistant (34, 37). We are in agreement with other studies that financial constraints (34, 43) are a hurdle to the treatment of AML in India and result in some of the patients not getting treated. We are also in agreement with Lima and colleagues (37), in which the results seen in specific biological subtypes of AML (cytogenetics or gene mutations) may not be identical to what has been seen in well-controlled western clinical trials. However, what is unique to our paper is that none of these studies has addressed assessment of immunophenotypic MRD in a real-world context.

Analogous to others (17–19, 22, 44), we found that the presence of MRD at early time points is highly predictive of relapse. We also document a slightly higher frequency of MRD-positive cases in our cohort as compared to others, perhaps reflecting suboptimal intensity of drugs administered or scheduling of chemotherapy cycles. We are in agreement with other published papers that MRD is an independent prognostic factor in predicting outcome. Importantly, it predicts relapse in cases that are in morphological remission and indicates that morphological assessment of remission is perhaps unreliable (27, 45). We found that MRD assessment may not offer additional information in favorable and poor-risk AML and yields maximum information in intermediate-risk cytogenetics group (22). We also demonstrate that there is a definitive advantage of MRD assessment in *NPM1* mutated AML. Here, the absence of MRD in even relatively not so favorable subsets for *NPM1*-positive *FLT3*-ITD-positive group predicted for a favorable outcome.

In our study, assessment of MRD was most useful in the intermediate cytogenetic risk group. Based on these data, we suggest that MRD-positive patients in the intermediate cytogenetic risk groups could perhaps be treated as high risk given their high potential to relapse. It has been published that nearly a quarter of AML patients who are MRD negative go on to relapse due to technical considerations, immunophenotypic shifts, or rapid emergence of chemoresistant clones (19, 22). Perhaps tracking of leukemia-specific mutations in the course of treatment using droplet digital PCR or next-generation sequencing will overcome the hurdles seen with immunophenotypic measurement of MRD.

Overall, our data seem to indicate that assessment of MRD in a resource-constrained setting adds much meaningful information supplementing conventional metrics such as cytogenetics and detection of mutations in *FLT3, NPM1*, and *CEBPA* genes. This information can be used for intensification of therapy for MRD-positive AMLs or de-intensification in MRD-negative AML patients. We hope that our results will add to the evidence that early assessment of AML MRD should be routinely done to guide post-remission treatment strategies.

## Data Availability

All datasets generated for this study are included in the manuscript and/or the Supplementary Files.

## Ethics Statement

This study was approved by the IEC III (project no 163) at ACTREC, Tata Memorial Centre. All the subjects were accrued only after proper informed consent and ethics approval by the institutional ethics committee.

## Author Contributions

NP: designed study, performed research, accrued the data, analyzed data, and wrote the paper. SHK: accrued the data. SKad, DS, AG, SP, AB, LN, HJ, BB, HM, MS, NK, and PS: accrued the data, analyzed data. CK, PB, SJ, SC, YB, SiG, ND, and GC: performed research. SKan, PT, and SuG: oversaw data analysis. PS and NP: designed study, performed research, accrued data, analyzed data, and wrote the paper.

### Conflict of Interest Statement

The authors declare that the research was conducted in the absence of any commercial or financial relationships that could be construed as a potential conflict of interest.
