# Supplementary material for: Utility of Immunophenotypic Measurable Residual Disease in Adult Acute Myeloid Leukemia—Real-World Context
Source: Front Oncol. 2019 Jun 13;9:450. doi: 10.3389/fonc.2019.00450 (PMC6584962; doi:10.3389/fonc.2019.00450)
Supplement: Supplementary file 1 [file Data_Sheet_1.docx]

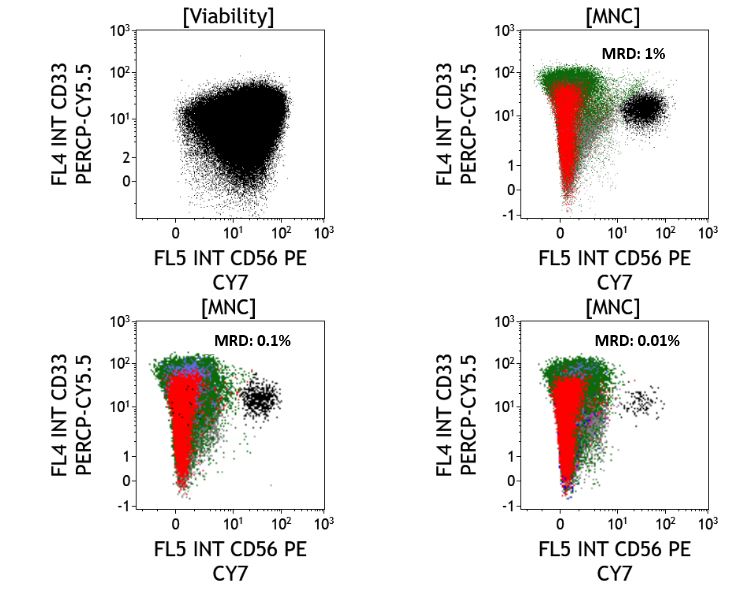


**Supplementary Figure 1: Limit of Detection experiment for the 8 colour FCM MRD assay. The plot on upper left represents the leukemic sample. The upper right plot shows dilution at 1%, the lower left and right plots show dilution at 0.1 and 0.01%.**

| **PI MRD Positive (n=108*)** | **Did Not Relapse (68)** | **Relapsed (40)** | **p value** |
| --- | --- | --- | --- |
| Favorable Cytogenetics | 22 | 11 | p=0.88 |
| Intermediate Cytogenetics | 36 | 19 |  |
| Poor Cytogenetics | 10 | 10 |  |
| *FLT3*-ITD Positive | 14 | 8 |  |
| *FLT3*-ITD Negative | 54 | 32 |  |
| *NPM1* Positive | 13 | 12 |  |
| *NPM1* Negative | 55 | 28 |  |
| *CEBPA* Positive | 5 | 2 |  |
| *CEBPA* Negative | 62 | 35 |  |
| **PI MRD Negative (n=191)** | **Did Not Relapse (151)** | **Relapsed (41)** |  |
| Favorable Cytogenetics | 55 | 14 | p=0.47 |
| Intermediate Cytogenetics | 86 | 20 |  |
| Poor Cytogenetics | 9 | 7 |  |
| *FLT3*-ITD Positive | 35 | 10 |  |
| *FLT3*-ITD Negative | 114 | 31 |  |
| *NPM1* Positive | 47 | 18 |  |
| *NPM1* Negative | 102 | 23 |  |
| *CEBPA* Positive | 14 | 3 |  |
| *CEBPA* Negative | 128 | 33 |  |

**Supplementary Table 1: Differences in characteristics of patients who were post induction MRD positive and relapsed/ post induction MRD positive and did not relapse as well as patients who were post induction MRD negative and relapsed/ post induction MRD negative and did not relapse. *One patient was excluded from this analysis as he died during the consolidation phase of therapy**
